# Pain relief and durable outcomes after revision reverse shoulder arthroplasty for failed primary arthroplasty

**DOI:** 10.1016/j.jsea.2026.100004

**Published:** 2026-02-19

**Authors:** Lorenz Fritsch, Mike Nocek, Marilee P. Horan, Grant J. Dornan, Brendon C. Mitchell, Mikalyn Defoor, Peter J. Millett

**Affiliations:** a The Steadman Philippon Research Institute, Vail, CO, USA; b Department of Sports Orthopaedics, Technical University of Munich, Munich, Germany; c The Steadman Clinic, Vail, CO, USA

**Keywords:** Revision reverse total shoulder arthroplasty, Failed shoulder arthroplasty, Patient-reported outcomes, Reoperation, Complications, Range of motion, Pain reduction

## Abstract

**Background:**

Outcomes following revision reverse total shoulder arthroplasty (revision rTSA) are variable, and the risk of re-revision remains considerable. The aim of the present study was to evaluate the clinical outcomes and outcome predictors after revision to rTSA following failure of a hemiarthroplasty (HA), anatomic total shoulder arthroplasty, or revision rTSA.

**Methods:**

After institutional review board approval, a retrospective review was performed of patients who underwent revision rTSA for failed HA, TSA, or rTSA between December 2005 and December 2020, with ≥24 months of follow-up. Pre-operative and post-operative patient-reported outcomes (American Shoulder and Elbow Surgeons [ASES], Quick Disabilities of the Arm, Shoulder, and Hand, Single Assessment Numeric Evaluation [SANE], SF-12 Physical Component Summary/Mental Component Summary, visual analog scale [VAS] pain, satisfaction) and range of motion were recorded. Relationships between subscapularis repair, humeral osteotomy, cement use, dominant-side surgery, and number of prior surgeries and final ASES, SANE, satisfaction, and pain were assessed. Subgroup analyses compared outcomes in patients with <5 years vs. ≥5 years of follow-up.

**Results:**

Of 41 eligible patients, 37 (90%) were available at final follow-up (25 male [68%], 12 female [32%]; mean age 63.5 ± 10.3 years; mean follow-up 82 ± 46 months). Index prostheses were HA (n = 17, 46%), anatomic total shoulder arthroplasty (n = 17, 46%), and reverse arthroplasty (n = 3, 8%). Indications for revision included rotator cuff failure (60%), infection (22%), instability (8%), aseptic glenoid loosening (5%), and refractory pain (5%). Significant improvements were observed in ASES (51.5 ± 11.1 to 72.1 ± 17.9; *P* < .001), Quick Disabilities of the Arm, Shoulder, and Hand (44.9 ± 11.8 to 34.7 ± 17.6; *P* < .001), daily pain VAS (3.3 ± 2.4 to 1.8 ± 2.1; *P* = .04), maximum pain VAS (7.7 ± 2.3 to 3.8 ± 3.2; *P* < .001), and SANE (42.5 ± 18.9 to 61.6 ± 26.6; *P* = .02). SF-12 Physical Component Summary/Mental Component Summary did not change (*P* > .05). Forward flexion improved significantly (*P* < .05). A greater number of previous surgeries correlated with lower final ASES (r_s_ = −0.43; *P* = .02). No other surgical variables were associated with outcomes. Short-term and long-term follow-up groups did not differ in function, pain, or satisfaction. Four patients (10.8%) underwent re-revision; 2 additional patients (5.4%) had complications (postoperative stiffness, acromial stress fracture).

**Conclusion:**

Revision rTSA leads to an improvement in patient-reported outcome measures and pain levels and demonstrates high durability at mid-term follow-up with low complication and failure rates. However, the gains in patient-reported outcome measures generally remain limited to a modest level. In addition, having undergone multiple prior surgeries may negatively affect the outcome. Future studies are needed to characterize the mid-term outcomes and durability of revision rTSA as this study is limited by its heterogeneity.

Reverse total shoulder arthroplasty (rTSA) has become an established surgical option for patients with cuff-deficient shoulder arthritis and complex shoulder pathology.^2^^,^^8^^,^^16^ When performed as a primary procedure, rTSA yields substantial improvements in pain relief, shoulder function, and range of motion. Mid-term studies report an excellent implant survival reaching 93% at 10 years.^6^^,^^10^^,^^17^^,^^18^^,^^26^

In the setting of failed anatomic total shoulder arthroplasty or hemiarthroplasty, conversion to revision rTSA has shown to significantly improve pain and functional outcomes.^3^^,^^9^ However, results remain inferior compared to primary rTSA, with higher complication and reoperation rates and revision rTSA carries the highest risk profile among shoulder arthroplasties.^20^^,^^25^ Although many patients experience pain relief and clinically meaningful functional improvement, complication rates have been reported as high as 32%, with similarly high re-revision rates.^6^^,^^26^^,^^32^ Implant failure, particularly baseplate loosening, periprosthetic fractures, and infection represent the most frequent causes of failure.^1^^,^^6^^,^^7^^,^^14^^,^^23^^,^^26^^,^^29^^,^^31^

Outcomes after re-revision rTSA are notably worse than after initial revision, with complications significantly reducing functional scores and patient satisfaction.^5^^,^^12^^,^^32^ Notably, the indication for revision rather than the type of primary implant (anatomic vs. reverse) may have a greater impact on clinical outcomes.^7^^,^^29^ Avoiding major complications remains critical for optimizing outcomes after revision rTSA.^27^^,^^32^

This study aims to assess outcomes, outcome predictors, and failure rates of revision rTSA after failed arthroplasty. Despite the inherently higher risk profile of revision rTSA, we hypothesize that it results in durable pain relief and sustained functional improvement over time, without a correspondingly high rate of implant failure.

## Methods

This retrospective review of prospectively collected data was conducted with approval from the WCG Institutional Review Board Clinical (WCG #20252021). All cases were performed by a single surgeon. Inclusion criteria included any patient who underwent revision rTSA following failed primary shoulder arthroplasty (including anatomic total shoulder arthroplasty, hemiarthroplasty, and rTSA) between December 2005 and December 2020. A minimum clinical follow-up of 24 months was required.

### Surgical technique

All revision procedures were performed with the patient positioned in a slightly inclined beach-chair position, with the operative arm stabilized using an electronic arm holder. A standard deltopectoral approach was utilized. Dissection was carried down to the joint capsule, and the subscapularis tendon was released via a peel-off technique. The existing prosthetic components were exposed and removed with care.

In cases where periprosthetic joint infection (PJI) was suspected, a prosthesis with antibiotic-loaded acrylic cement spacer was implanted as part of a two-staged procedure, and definitive revision rTSA was delayed until final culture results were available and the infection had been eradicated.

Preparation of the glenoid and humeral canal was carried out according to standard technique. Structural bone grafting of the glenoid was employed selectively in cases of severe bone loss when absolutely necessary. In the event that the prosthesis could not be removed, a humeral osteotomy was performed. In some instances, drilling was performed to remove bone sclerosis prior to reaming. An appropriately sized glenosphere was implanted, and a press-fit humeral stem with matching cup and inlay components was used. Following prosthetic reduction, intraoperative stability was thoroughly assessed. The subscapularis tendon was repaired using a modified Mason-Allen transosseous technique. A surgical drain was placed, and layered wound closure was performed in standard fashion.

### Rehabilitation

Post-operatively, patients were immobilized in a shoulder abduction sling for three weeks and permitted early passive range of motion. Active and active-assisted motion was initiated at approximately four weeks post-operatively. External rotation was restricted to 30° for the first 3 to 4 weeks to protect the subscapularis repair.

### Outcome measurement

Patient-reported outcome measures (PROMs) were collected pre-operatively and at final follow-up post-operatively (minimum of two years) using an electronic database.

Primary outcomes included the American Shoulder and Elbow Surgeons (ASES) score, the minimal clinically important difference (MCID) for the ASES score, and the patient acceptable symptom state (PASS) for the ASES score. Recent literature has quantified the anchor-based ASES MCID and PASS thresholds after first revision rTSA as 20.1 and 63.5, respectively.^12^ Additional PROMs collected included the short version of the Disabilities of the Arm, Shoulder and Hand (QuickDASH) questionnaire, the Single Assessment Numeric Evaluation (SANE), the Physical Component Summary and the Mental Component Summary of the 12-Item Short-Form Health Survey (SF-12), a visual analog scale for pain (maximum and daily score), and a subjective satisfaction score on a 10-point Likert scale, with 10 representing maximum satisfaction. Range of motion was assessed pre-operatively and at the most recent follow-up examination (which was performed at least four months after surgery). Furthermore, the effects of subscapularis repair, humeral osteotomy, cement use, surgery on the dominant shoulder, and the number of previous surgeries on the ASES score, SANE score, patient satisfaction, and pain at follow-up were assessed. Incomplete PROM data were excluded from the analysis.

A subgroup analysis of the assessed PROMs was conducted to compare patients for whom follow-up data were available for less than 5 years with those for whom follow-up extended beyond 5 years. Secondary outcomes recorded include the incidence of complications and failures. Failure was defined as any secondary revision surgery requiring direct intervention to the revision rTSA construct, including glenoid/humeral component exchange or complete arthroplasty explanation. Additional surgical procedures not involving the prosthesis itself, such as arthroscopic interventions, were classified as complications rather than failures.

### Statistical analysis

Statistical analysis was conducted using SPSS software (version 30.0; IBM Corp., Armonk, NY, USA). Categorical variables are presented as counts and percentages. Normality of continuous data was assessed using the Kolmogorov–Smirnov test and confirmed through visual inspection. Normally distributed variables are reported as mean with standard deviation, while non-normally distributed variables are presented as median with interquartile range (25th-75th percentile). Group comparisons were performed using the Student *t*-test for normally distributed variables and the Wilcoxon rank-sum test for nonparametric data. Within-subjects comparisons of baseline and post-operative PROMs were compared using paired *t*-tests or the Wilcoxon signed-rank test. Spearman correlation analysis was used to evaluate associations between continuous variables. Statistical significance was defined as a *P* value <.05.

## Results

### Patient demographics and surgical data

Between December 2005 and December 2020, 37 of 41 patients (90.2%) were available for final analysis. Thirty-one patients (84%) received their primary treatment elsewhere. In 12 cases (32%), a two-stage procedure was performed. A detailed overview of the demographic and surgical results is presented in Table I.

**Table I:** Patient demographics and surgical data.

Variable	Value
Mean age at surgery (yr)	63.5 ± 10.3
Mean follow-up (mo)	82 ± 45.7
Time from initial arthroplasty to revision rTSA (mo)	39.6 ± 45.2
Sex distribution	25 males (67.6), 12 females (32.4)
Dominant shoulder (n (%))	23 (62.2)
Number of previous surgeries	2 (1-3)
Type of arthroplasty	
Hemiarthroplasty (n (%))	17 (45.9)
Anatomic total shoulder arthroplasty (n (%))	17 (45.9)
Reverse total shoulder arthroplasty (n (%))	3 (8.2)
Revision indications	
Rotator cuff failure (n (%))	22 (59.5)
Infection (n (%))	8 (21.6)
Instability (n (%))	3 (8.1)
Persistent pain (n (%))	2 (5.4)
Aseptic glenoid loosening (n (%))	2 (5.4)
Humeral osteotomy performed (n (%))	9 (24.3)
Subscapularis repair (n (%))	10 (27.0)
Cement use (n (%))	14 (37.8)

*rTSA*, reverse total shoulder arthroplasty.

Percentages are reported relative to the total cohort (n = 37). Data following a normal distribution are presented as mean ± standard deviation, whereas non-normally distributed data are presented as median and interquartile range.

### Patient-reported outcome measures

The mean ASES score (*P* < .001), QuickDASH score (*P* < .001), and daily pain (*P* = .04) significantly improved from pre-operative to minimum 2-year post-operative. Maximum pain levels were also significantly reduced (*P* < .001). The SANE score demonstrated significant improvement as well (*P* = .02). In contrast, no improvement was observed in the SF-12 Mental Component Summary or Physical Component Summary scores (*P* > .05). A detailed overview of the PROMs is presented in Table II.

**Table II:** Patient-reported outcome measures.

Outcome measure	Pre-operative	Post-operative (minimum 2-yr)	*P* value
ASES	51.5 ± 11.1	72.1 ± 19.7	**<.001**
QuickDASH	44.9 ± 11.8	34.7 ± 17.6	**<.001**
Daily pain VAS	3.3 ± 2.4	1.8 ± 2.1	**.04**
Maximum pain VAS	7.7 ± 2.3	3.8 ± 3.2	**<.001**
SANEscore	42.5 ± 18.9	61.6 ± 26.6	**.02**
Patient satisfaction (final FU)	N/A	6.6 ± 2.9	
SF-12 MCS	51.9 ± 10.1	55.8 ± 6.1	.12
SF-12 PCS	41.4 ± 8.0	42.0 ± 10.3	.62

*ASES*, American Shoulder and Elbow Surgeons; *QuickDASH*, Quick Disabilities of the Arm, Shoulder, and Hand; *VAS*, visual analog scale; *SANE*, Single Assessment Numeric Evaluation; *SF-12 MCS*, 12-Item Short-Form Health Survey Mental Component Summary; *SF-12 PCS*, 12-Item Short-Form Health Survey Physical Component Summary; *FU*, follow-up.

Normally distributed continuous variables are shown as mean ± standard deviation. Significant *P* values are marked in bold letters.

Overall, 16 patients (43%) reached the MCID for the ASES score. In total, 21 patients (57%) achieved the PASS for the ASES score.^12^ External rotation did not significantly alter from pre-operative to post-operative assessment (35.5 ± 20.4° vs. 44.8 ± 18.5°; *P* = .20). A significant improvement was observed in active flexion, increasing from 83.6 ± 38.6° pre-operatively to 142.6 ± 23.6° post-operatively (*P* < .001).

None of the factors—dominant shoulder involvement, humeral osteotomy, cement use, or subscapularis repair—showed a significant effect on outcomes. However, a higher number of prior surgeries was associated with lower ASES scores (r_s_ = −0.43, *P* = .02).

### Subgroup analysis

Although ASES, QuickDASH, and SANE scores improved significantly from pre-operative to postoperative assessments, no additional significant differences in outcomes were observed between patients with short-term follow-up (<5 years) and those with mid-term follow-up (≥5 years). The subgroup analysis can be found in Table III.

**Table III:** Subgroup analysis (short-term vs. mid-term follow-up).

Outcome measure	Short-term FU	Mid-term FU	*P* value
ASES	64.3 ± 21.5	71.8 ± 18.9	.33
QuickDASH	39.2 ± 20.8	34.7 ± 18.4	.53
Daily pain	2.1 ± 2.3	1.6 ± 2.0	.52
Maximum pain	4.3 ± 3.3	3.6 ± 3.3	.57
SANE score	50.4 ± 30.9	67.1 ± 21.7	.09
SF-12 PCS	41.3 ± 11.1	42.5 ± 11.4	.08
SF-12 MCS	54.8 ± 7.5	54.5 ± 7.7	.90
Patient satisfaction	7.5 ± 2.9	5.7 ± 3.1	.11

*ASES*, American Shoulder and Elbow Surgeons; *QuickDASH*, Quick Disabilities of the Arm, Shoulder, and Hand; *SANE*, Single Assessment Numeric Evaluation; *SF-12 PCS*, 12-Item Short-Form Health Survey Physical Component Summary; *SF-12 MCS*, 12-Item Short-Form Health Survey Mental Component Summary; *FU*, follow-up.

Subgroup analysis comparing those with only short-term follow-up (<5 years) to those with mid-term follow-up (≥5 years). Normally distributed continuous variables are shown as mean ± standard deviation.

### Complications and failures

In total, 4 patients (10.8%) required a second revision rTSA surgery. One patient developed a deep venous thrombosis of the operative shoulder in the immediate post-operative period, followed by PJI. A second patient required revision because of PJI associated with glenoid loosening. Both patients had a history of PJI after their index arthroplasty and both cases required a two-staged second revision rTSA after infection eradication. No cases of PJI were reported in patients without a history of PJI at the index procedure. Two additional patients underwent revision revision rTSA. One patient with aseptic glenoid loosening was treated with reimplantation of a revision rTSA, and one patient with dislocation was managed with revision to a larger humeral stem and larger humeral inlay.

A total of 2 patients (5.4%) reported nonrevision complications. One patient developed a painful post-operative contracture that was successfully treated with arthroscopic arthrolysis, ultimately resulting in clinical improvement. Another patient sustained a stress fracture of the acromion while lifting a heavy object, which was successfully managed with conservative treatment.

## Discussion

Consistent with our initial hypothesis, revision rTSA was associated with improvements in PROMs, forward flexion, and pain relief. Furthermore, revision rTSA was found to be a durable procedure with relatively low rates of complications and implant failure at mid-term follow-up. However, improvement in mean patient-reported outcomes remained modest, and not all patients achieved the MCID. Furthermore, undergoing multiple surgeries before revision rTSA decreases the likelihood of a beneficial outcome.

The outcomes of this study are consistent with previous reports on revision rTSA and support its beneficial effect. Although other studies have reported similar findings, revision rTSA continues to yield significantly inferior functional and predictable outcomes compared to primary rTSA.^4^^,^^11^^,^^12^^,^^21^ Whereas ASES scores in primary rTSA range from 69.1 to 80.8,^4^^,^^15^^,^^21^ and SANE scores range from 69.1 to 80.8,^4^^,^^21^ revision rTSA demonstrates markedly inferior outcomes, with ASES scores ranging from 63.5 to 70.1,^11^^,^^21^ and SANE scores around 67.8.^11^^,^^21^

Importantly, patients in this series achieved significant improvements in both PASS and MCID on average. However, when analyzing the individual PASS and MCID achievements, the number of patients accomplishing these goals occurred in ∼50% of patients. These finding are similar to those reported by Hao KA et al.^12^ They found that 42% patients undergoing revision rTSA achieved the MCID for ASES and 53% achieved the PASS for ASES at a minimum of 24 months.^12^ The expected success of primary rTSA is more predictable with 88.8% of patients achieving the MCID for ASES and 65% of patients achieving the PASS threshold for ASES at 5-year follow-up.^4^ These differences highlight the challenges and complexity of performing revision rTSA.^4^^,^^13^^,^^28^ This study highlights the durability of revision rTSA, with complication and failure rates similar to primary rTSA. In our cohort, we found a 5.4% nonrevision complication rate and 10.8% failure rate. Primary rTSA complication rates have been reported to range from 9.4% to 22%.^10^^,^^19^ In the few studies reporting on revision rTSA outcomes, complication rates have ranged between 23% and 35%.^14^^,^^32^ Failure rates in primary rTSA have been reported to occur at a range of 2.5%-5.8%, being slightly lower than in our cohort.^10^^,^^24^ Rates of failure for revision rTSA have previously been reported to occur at much higher rates, ranging from 22% to 32%, with implant survivorship free from further revision at 5 years between 75% and 85%.^5^^,^^14^^,^^22^^,^^25^^,^^30^ These differences may be accounted for by quite young age at time of the surgery. Similar to our cohort, the most common previously described reasons for implant failure after revision rTSA include dislocation, baseplate loosening, and infection.^5^^,^^22^

Taking these data into account, revision rTSA can be performed and may be offered as a salvage option with a moderate risk profile to improve functional outcomes in cases where primary procedures fail.

### Limitations

This study has several limitations. First, its retrospective design introduces the potential for selection and reporting bias. Second, owing to its single-center study design, the study lacks external validity. Third, functional outcomes were not comprehensively assessed, as range of motion was not systematically evaluated. Fourth, follow-up duration varied considerably across patients, ranging from short-term to mid-term, which may have influenced the consistency of outcomes. Furthermore, although statistically significant changes were observed, the outcomes remain moderate.

Also, an important limitation of this study is the heterogeneous nature of the primary arthroplasty procedures and the diverse indications leading to conversion.

Furthermore, only external rotation and forward flexion were included in the analysis, as other range-of-motion parameters were inconsistently reported across the datasets.

In addition, the comparative analyses between groups were limited by reduced statistical power, as indicated by the post hoc power analysis. Furthermore, all cases were performed by a single surgeon, which may limit the generalizability of the data, but also controls for surgeon variability. In contrast, the follow-up period may have led to the observation of a learning-curve effect among the practicing surgeon. Furthermore, as a sports medicine referral clinic, our patients tend to have fewer comorbidities, which could significantly influence the outcomes. Finally, radiographic assessments were not performed, restricting our ability to evaluate implant positioning and potential structural changes following revision rTSA. Due to the lack of radiographic assessment, some failures may not have been detected, and important information regarding the arthroplasty may have been missed.

## Conclusion

Revision rTSA leads to an improvement in PROMs as well as pain levels and demonstrates high durability at long-term follow-up with low complication and failure rates. However, the gains in PROMs generally remain limited to a modest level. In addition, having undergone multiple prior surgeries may negatively affect the outcome. Future studies are needed to characterize the mid-term outcomes and durability of revision rTSA, as this study is limited by its heterogeneity.

## Disclaimers:

Funding: No funding was disclosed by the authors.

Conflicts of interest: Lorenz Fritsch discloses research grant support from AGA,; grant sponsored by Arthrex; not related to this work. Any additional authors, their immediate families, and any research foundations with which they are affiliated have not received any financial payments or other benefits from any commercial entity related to the subject of this article.
